# Giant laryngeal polyp: an unusual indication of tracheostomy

**DOI:** 10.11604/pamj.2017.26.76.11784

**Published:** 2017-02-20

**Authors:** Moncef Sellami, Mohamed Amine Chaabouni

**Affiliations:** 1 Department of Oto-rhino-laryngology, Head and Neck Surgery, Habib Bourguiba University Hospital, Sfax, Tunisia; 2 Sfax Medical School, University of Sfax, Sfax, Tunisia

**Keywords:** Airway obstruction, larynx, polyp, surgical treatment

## Image in medicine

Benign laryngeal lesions, especially laryngeal polyps are very common and they usually only cause hoarseness and rarely develop into dyspnea. This condition is generally attributed to smoking. Cardiorespiratory failure and sudden death due to large laryngeal polyp was reported in the literature. The preferred treatment of large polyps is surgical excision using suspension microlaryngoscopy. We present a case of a 60-year-old man presented to the emergency department with stridor and severe dyspnea. He had a 4-month history of a neglected inspiratory dyspnea and dysphonia. Fiberoptic laryngoscopy revealed a large laryngeal mass causing airway obstruction. Administration of oxygen, inhalation and intravenous administration of corticosteroid did not relieved his symptoms. As intubation was judged impossible, an emergency tracheostomy was performed to salvage the patient. After tracheostomy, the dyspnea ceased. Computed tomography scan showed a homogeneous regular formation of 2.5×1.5 cm obstructing the glottis and the subglottis lumen. Direct laryngoscopy under general anesthesia identified a valve-like large polyp attached to the anterior wall of the subglottic region. The polyp was totally excised with no complication. Decannulation was carried out on the second postoperative day. The mass was histologically diagnosed as normal vocal cord polyp.

**Figure 1:**
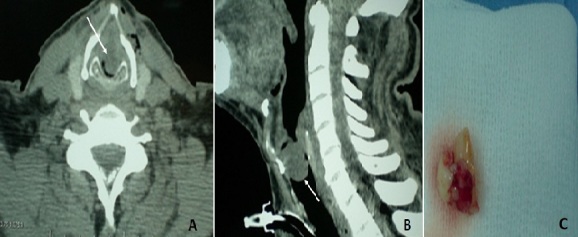
A) axial computed tomography image showing a huge subglottic mass (arrow); B) sagittal computed tomography image showing a homogeneous regular obstructive formation obstructing the glottis and subglottis lumen (arrow); C) excision of the large polypoid mass

